# Fragility Index analysis for robustness of evidence in Randomized Controlled Trials in National Comprehensive Cancer Network (NCCN) guidelines for rectal cancer

**DOI:** 10.1002/ijc.35521

**Published:** 2025-06-23

**Authors:** Nir Horesh, Justin Dourado, Peter Rogers, Pauline Aeschbacher, Zoe Garoufalia, Rachel Gefen, Ebram Salama, Sameh Hany Emile, Steven D. Wexner

**Affiliations:** ^1^ Ellen Leifer Shulman and Steven Shulman Digestive Disease Center Cleveland Clinic Florida Weston Florida USA; ^2^ Department of Surgery and Transplantations Sheba Medical Center, Ramat Gan and Faculty of Medicine, Tel Aviv University Tel Aviv Israel; ^3^ Florida Atlantic University, College of Medicine Department of General Surgery Boca Raton FL USA; ^4^ Department of Visceral Surgery and Medicine Inselspital, Bern University Hospital, University of Bern Switzerland; ^5^ Department of General Surgery Hadassah Medical Organization and Faculty of Medicine, Hebrew University of Jerusalem Jerusalem Israel; ^6^ Faculty of Medicine, Colorectal Surgery Unit Mansoura University Mansoura Egypt

**Keywords:** immunotherapy, rectal cancer, surgery, survival

## Abstract

Robustness of evidence from randomized controlled trials (RCTs) is crucial for guiding clinical decisions in rectal cancer. We evaluated the reliability of RCTs cited by the National Comprehensive Cancer Network (NCCN) guidelines for rectal cancer using the Fragility Index (FI) that quantifies the stability of trial outcomes. RCTs referenced in the latest NCCN guidelines for rectal cancer were reviewed. Data from eligible trials were extracted. FI was calculated to assess the robustness of evidence across different treatment modalities. Sixty‐seven RCTs (published: 1987–2022) involving 16,990 patients were analyzed. Most studies (58.2%) were conducted in Europe. Common treatment areas included metastatic liver disease (28.9%) and neoadjuvant chemotherapy (14.9%). Primary outcomes were disease‐free survival and overall survival (OS) in 15 studies each (22.4%), local recurrence rates in 6 (9%), and tumor response in 5 (7.5%). The median FI was 9 (interquartile range [IQR] 2–20). Studies on surgical interventions had the highest median FI (21 [IQR 7–27]) followed by studies on neoadjuvant radiotherapy (19 [IQR 14–25]). Neoadjuvant immunotherapy studies had the lowest median FI of 0, indicating less robust evidence. Notably, surgical intervention studies showed the largest gap between FI and patients lost to follow‐up (21 vs. 13.5), while neoadjuvant immunotherapy studies showed more patients lost to follow‐up than the median FI (0 vs. 5), highlighting the need for stronger evidence. In conclusion, evidence supporting most treatments for rectal cancer in the NCCN guidelines is robust, although neoadjuvant immunotherapy requires further scrutiny due to its low FI. FI offers a nuanced perspective on the reliability of trial outcomes.

## Graphical abstract

What's new?

Data from randomized controlled trials (RCTs) forms a foundation for rectal cancer treatment guidelines. The reliability of such data is therefore essential for evidence‐based clinical decision‐making. This study assessed the robustness of RCTs cited in the latest National Comprehensive Cancer Network (NCCN) guidelines for rectal cancer using the Fragility Index (FI), which quantifies the stability of findings. Analyses show that surgical intervention and neoadjuvant radiotherapy trials are highly robust. By contrast, neoadjuvant immunotherapy trials exhibited a median FI of 0, suggesting highly fragile outcomes. The findings indicate that while most NCCN‐cited trials provide stable evidence, emerging modalities warrant further investigation.

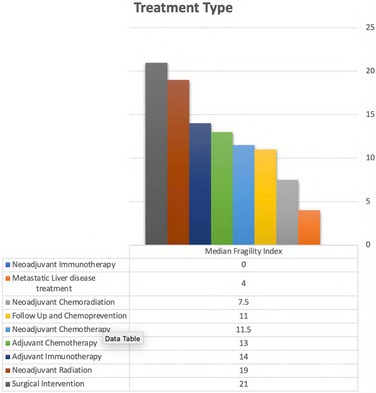

**Abbreviations**
ACG: American College of Cardiology
AAP: advanced adenomatous polyps
DFS: disease‐free survival
FI: Fragility Index
HR: hazard ratio
IQR: interquartile range
NCCN: National Comprehensive Cancer Network
OS: overall survival
PFS: progression‐free survival
PRISMA: Preferred Reporting Items for Systematic Reviews and Meta‐Analyses
RCT: randomized controlled trial
TME: total mesorectal excision

## 1 INTRODUCTION

Rectal cancer is a significant global health challenge. In the United States alone, it accounts for over 50,000 annual new diagnoses.^1^ The treatment paradigms for rectal cancer have evolved; advances in surgical techniques, combined with the integration of neoadjuvant and adjuvant therapies, have revolutionized patient outcomes.^2^ These shifts highlight the importance of evidence‐based guidelines to ensure optimal patient care.

The National Comprehensive Cancer Network (NCCN) guidelines are a global cornerstone.^3^ They NCCN guidelines offer a comprehensive framework for treating various malignancies, including rectal cancer, based on rigorous reviews of available evidence, primarily from randomized controlled trials (RCTs).^4^ The NCCN guidelines adopt an integrative approach, combining insights from surgical, medical, and radiation oncology, ensuring patients receive a holistic and state‐of‐the‐art therapeutic regimen. Although these guidelines are established in the United States, a recent survey in low‐ and middle‐income countries showed that despite the need for treatment modifications occasionally due to lack of resources, 92% of oncologists in these regions continue to rely on the NCCN guidelines, underscoring their importance and influence in guiding oncological care worldwide.^5^

However, the robustness of the evidence underpinning these guidelines is crucial. The Fragility Index (FI), introduced by Walsh et al. in 2014, is a statistical tool that assesses the robustness of RCT results and quantifies how many events would need to change for a statistically significant result to become non‐significant.^6^ A low FI suggests that results are “fragile” and could be influenced by minor data alterations.^7^, ^8^ Conversely, high FI indicates robust findings.^9^ The FI's utility extends beyond statistical analysis; it critically evaluates the robustness of RCTs in various therapeutic areas. For instance, some “practice‐changing” RCTs in breast cancer had surprisingly low FIs, leading to a reevaluation of their impact on clinical guidelines.^10^

Given the pivotal role of the NCCN guidelines and the increasing use of the FI, this study examined the evidence underpinning the RCTs cited in the NCCN guidelines for rectal cancer. We sought to determine the robustness of this evidence by assessing the FI of these studies. By doing so, we aimed to offer clinicians and researchers insights into the strength and reliability of the evidence shaping current treatment recommendations, ensuring that clinical decisions are based on robust and reliable data.

## 2 METHODS

This meta‐analysis was conducted in accordance with the Preferred Reporting Items for Systematic Reviews and Meta‐Analyses (PRISMA) guidelines.^11^ The references list of the most recent version of the NCCN guidelines for rectal cancer (version 4.2023—July 25, 2023)^12^ were reviewed by two authors (JD, PR) to identify the RCTs that were cited and used to support the recommendations of the guidelines.

### 2.1 Inclusion and exclusion criteria

We considered RCTs included in the latest version of the NCCN treatment guidelines for rectal cancer. These studies needed to have clear results showing statistical significance for either primary or secondary binary outcomes. Additionally, it was essential for these studies to either mention their sample size outright or have a power calculation. Lastly, the structure of these studies had to have been based on a parallel arm design. Only studies with a clearly defined primary outcome amenable to FI calculation (binary primary outcome) were included, so that the FI can be accurately determined and is meaningful in the context of the study's findings.

We excluded duplicate studies to prevent data redundancy. In addition, we excluded reviews and pooled analyses. In cases where RCTs had subsequent versions with more comprehensive outcomes, we prioritized the most recent version and excluded the earlier iterations. Studies that compared more than two groups were also omitted to maintain clarity and consistency in our analysis. Finally, any study that failed to provide data on the primary outcomes was not considered.

### 2.2 Data collection process

Data extraction was systematically conducted by two independent reviewers (JD and PR). We used a standardized data extraction form to gather relevant information from each eligible RCT. This form included details related to the primary outcome such as the number of events and non‐events in each arm, p‐value, and associated statistical tests. Additionally, they recorded other pertinent details such as the study's objectives, interventions compared, and patient populations. Any disagreements between the two reviewers were resolved through discussion and, if necessary, by consulting a third reviewer (SE) to reach a consensus.

### 2.3 Statistical analysis

The FI for each RCT was calculated using the online tool available at https://clincalc.com/Stats/FragilityIndex.aspx.^13^ The FI was calculated by iteratively modifying the number of events in the treatment or control group until the statistical significance (set at *p* < .05) of the result was lost. Statistical analyses were conducted using EZR (version 1.55) and R software (version 4.1.2).^14^ For continuous data, if the distribution was normal, values were expressed as mean and standard deviation. In cases where the distribution was not normal, values were presented as median and interquartile range (IQR). Depending on the distribution, either the Student *t*‐test or the Mann–Whitney *U* test was used to analyze continuous variables.

## 3 RESULTS

**FIGURE 1:**
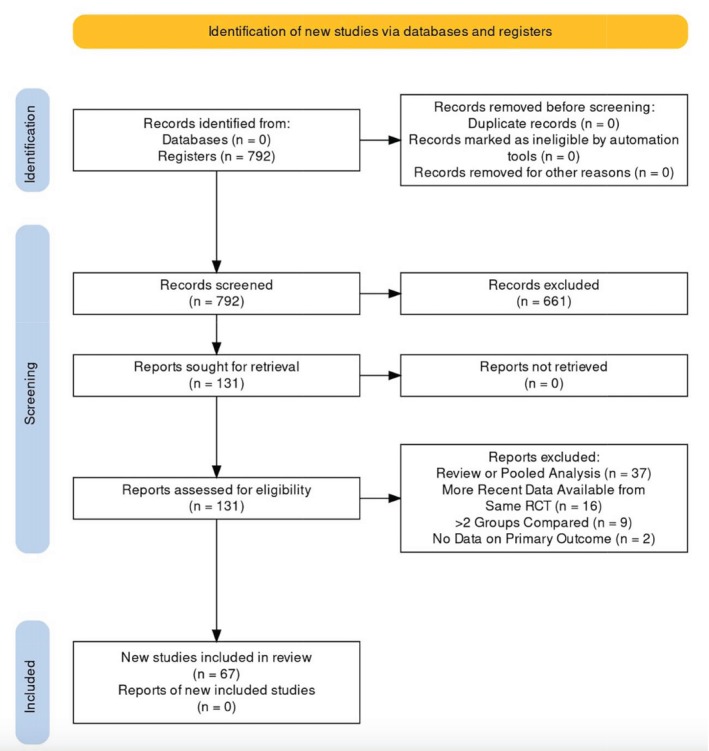
Study flowchart.

**FIGURE 2:**
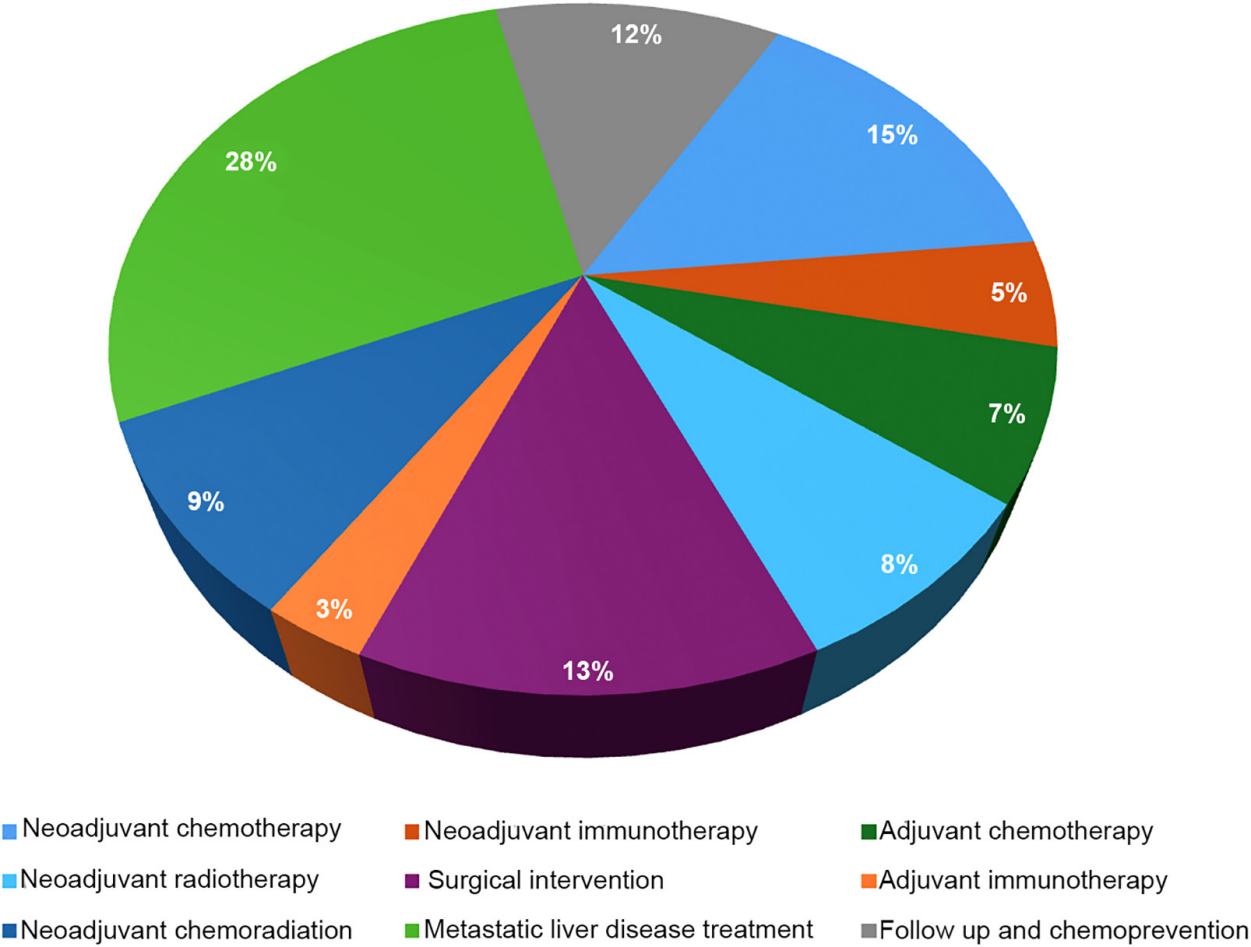
Main focus of included studies.

Overall, the analysis included a total of 67 studies including 16,990 patients were reported. A PRISMA flowchart of included studies is shown in Figure 1. The median number of patients in each study was 161 (IQR 72–255). The studies were conducted from 1987 to 2022, with a mean study duration of 4.8 years (SD 2). Thirty‐nine (58.2%) studies were conducted in Europe, 6 (9%) in America, 10 (14.9%) in China, 10 (14.9%) were multinational collaborations, and 2 (3%) were from other countries. The primary outcome of the studies was overall survival (OS) (15 studies, 22.4%), disease‐free survival (DFS) (15 studies, 22.4%), progression‐free survival (PFS) (6 studies; 9%), and tumor response (5 studies, 7.5%). Characteristics of the included studies are detailed in Table 1. The most assessed treatment type was therapy for metastatic liver disease (19 studies, 28.3% of the total studies). Neoadjuvant chemotherapy was assessed in 10 studies (14.9%), while surgical interventions were the focus of 9 studies (13.4%). There were six studies (9%) on neoadjuvant chemoradiation and five on adjuvant chemotherapy (7.5%). Additionally, five studies explored the potential of neoadjuvant radiotherapy and three studies examined neoadjuvant immunotherapy. Finally, eight studies were centered around follow‐up and chemoprevention strategies (11.9%) (Figure 2).

**TABLE 1:** Characteristics of the included studies.

Treatment type	Study	Journal	Short name	Public. year	Country	Setting	Primary outcome
Neoadjuvant chemotherapy	Conroy et al.^15^	Lancet Oncol	PRODIGE 23	2021	France	Multicenter	DFS at 3 years
	Hofheinz et al.^16^	Lancet Oncol	NR	2012	Germany	Multicenter	OS at 5 years
	Rodel et al.^17^	Lancet Oncol	CAO/ARO/AIO‐04	2015	Germany	Multicenter	DFS at 3 years
	Marechal et al.^18^	Ann Oncol	NR	2012	Belgium	Multicenter	ypT0‐1N0 rate
	Fokas et al.^19^	JAMA Oncol	CAO/ARO/AIO‐12	2022	Germany	Multicenter	DFS at 3 years
	Aschele et al.^20^	J Clin Oncol	STAR‐01	2011	Italy	Multicenter	ypCR
	Gerard et al.^21^	J Clin Oncol	ACCORD 12/0405 PRODIGE 2	2012	France	Multicenter	DFS at 3 years
	Fernandez‐Martos et al.^22^	JAMA Oncol	GEMCAD 1402	2019	Spain	Multicenter	ypCR
	Bujko et al.^23^	Br J Surg	NR	2006	Poland	Multicenter	DFS at 4 years
	Fernandez‐Martos et al.^24^	Ann Oncol	GCR‐3	2015	Spain	Multicenter	DFS at 5 years
Neoadjuvant radiotherapy	Sebag‐Montefiore et al.^25^	Lancet	MRC CR07/NCIC‐CGT C016	2009	UK/South Africa/New Zealand/Canada	Multicenter	Local recurrence
	Van Gjin et al.^26^	Lancet Oncol	NR	2011	Europe/Canada	Multicenter	Local recurrence
	Francois et al.^27^	J Clin Oncol	Lyon R90‐01	1999	France	Multicenter	Sphincter preserving surgery
	Ngan et al.^28^	J Clin Oncol	TROG 01.04	2012	Australia/New Zealand	Multicenter	Local recurrence
	Latkauskas et al.^29^	BMC Cancer	NR	2016	Lithuania	Single center	ypCR
Neoadjuvant chemoradiation	Sauer et al.^30^	J Clin Oncol	CAO/ARO/AIO‐94	2012	Germany	Multicenter	OS at 10 years
	Lefevre et al.^31^	Ann Surg	Greccar‐6	2016	France	Multicenter	ypCR
	Cisel et al.^32^	Ann Oncol	Polish II	2019	Poland	Multicenter	Radical Sx rate
	Jin et al.^33^	J Clin Oncol	STELLAR	2022	China	Multicenter	DFS at 3 years
	Bahadoer et al.^34^	Lancet Oncol	RAPIDO	2021	Europe/USA	Multicenter	Disease‐related treatment failure at 3 years
	Bujko et al.^35^	Ann Oncol	NR	2016	Poland	Multicenter	R0 resection rate
Neoadjuvant immunotherapy	Dewdney et al.^36^	J Clin Oncol	EXPERT‐C	2012	Europe	Multicenter	Complete response
	Helbling et al.^37^	Ann Oncol	SAKK 41/07	2013	Switzerland, Hungary	Multicenter	pNC/CR
	Borg et al.^38^	Clin Colorectal Cancer	INOVA	2019	France	Multicenter	pCR
Surgical intervention	Jeong et al.^39^	Lancet Oncol	COREAN trial	2014	Korea	Multicenter	DFS at 3 years
	Chen et al.^40^	Surg Laparosc Endosc Tech	NR	2015	China	Single center	Complications
	Bonjer et al.^41^	N Engl J Med.	NR	2015	Multinational	Multicenter	LRR at 3 years
	Jayne et al.^42^	J Clin Oncol	UK MRC CLASICC	2007	UK	Multicenter	OS at 3 years
	Fleshman et al.^43^	Ann Surg	ACOSOG Z6051	2019	USA/Canada	Multicenter	DFS at 2 years
	Stevenson et al.^44^	Ann Surg	ALaCaRT	2019	Australia/New Zealand	Multicenter	LRR at 2 years
	Araujo et al.^45^	Rec Hosp Clin Fac Med Sao Paulo	NR	2003	Brazil	Single center	LRR
	Jayne et al.^46^	JAMA	ROLARR	2017	Multinational	Multicenter	Conversion
	Kim et al.^47^	Ann Surg	NR	2018	South Korea	Single center	TME quality
Metastatic liver disease treatment	Ghirinchelli et al^48^	J Cancer Res Clin Oncol	HEARTO	2019	France	Multicenter	PFS at 6 months
	Martin et al.^49^	Cancer	NR	2015	USA	Multicenter	Response rate
	Lammer et al.^50^	Cardiovasc Intervent Radiol	PRECISION V	2010	Europe	Multicenter	Tumor response at 6 months
	van Malenstrein et al.^51^	Onkologie	NR	2011	Belgium	Single center	Tumor response
	Mulcahy et al.^52^	J Clin Oncol	NR	2021	Multinational	Multicenter	PFS/hPFS
	van Hazel et al.^53^	J Clin Oncol	SIRFLOX	2016	Multinational	Multicenter	PFS
	Palma et al.^54^	J Clin Oncol	SABR‐COMET	2020	Multinational	Multicenter	OS
	Tang et al.^55^	J Clin Oncol	BECOME	2020	China	Single center	R0 resection
	Ye et al.^56^	J Clin Oncol	NR	2013	China	Single center	Conversion to resection
	Saltz et al.^57^	J Clin Oncol	NR	2008	Multinational	Multicenter	PFS
	Modest et al.^58^	J Clin Oncol	VOLFI	2019	Germany	Multicenter	Objective response rate
	Souglakos et al.^59^	Br J Cancer	NR	2006	Greece	Multicenter	OS at 2 years
	Nordlinger et al.^60^	Lancet Oncol	EORTC 40983	2013	Multinational	Multicenter	PFS
	Masi et al.^61^	J Natl Cancer Inst	NR	2011	Italy	Multicenter	OS at 2 years
	Folprecht et al.^62^	Lancet Oncol	CELIM	2010	Germany/Austria	Multicenter	Tumor response
	Bridgewater et al.^63^	Lancet Oncol	New EPOC	2020	UK	Multicenter	PFS
	Kanemitsu et al.^64^	J Clin Oncol	iPACS	2021	Japan	Multicenter	OS
	Moulton et al.^65^	JAMA	NR	2014	Canada	Multicenter	Change in surgical management
Adjuvant chemotherapy	Feng et al.^66^	Oncotarget	NR	2016	China	Multicentre	3‐year DFS
	Wolmark et al.^67^	J Nat Cancer Inst	NR	2000	USA	Multicentre	8‐year relapse‐free survival
	Aldo et al.^68^	Radiot Oncol	(I‐CNR‐RT)	2014	Italy	Multicentre	5‐year OS
	Breugom et al.^69^	Ann Oncol	PROCTOR‐SCRIPT	2014	The Netherlands and Sweeden	Multicentre	5‐year OS
	Hong et al.^70^	J Clin Oncol	ADORE	2019	Korea	Multicentre	6‐year DFS
Adjuvant immunotherapy	Chakravarthy et al.^71^	Oncologist	E5204	2020	USA	Multicentre	5‐year OS
	Snoeren et al.^72^	Neoplasia	HEPATICA	2017	The Netherlands	Multicentre	2‐year DFS
Follow‐up and chemoprevention	Hennink et al.^73^	J Clin Oncol	NR	2015	The Netherlands	Multicenter	Advanced adenomatous polyps (AAP) detection
	Urashima et al.^74^	JAMA	AMATERASU	2019	Japan	Single centre	DFS 5 years
	Pietra et al.^75^	DCR	NR	1998	Italy	Single centre	OS at 5 years
	Rodriguez‐Moranta^76^	J Clin Oncol	NR	2006	Spain	Multicenter	OS at 5 years
	Secco et al.^77^	Eur J Surg Onc	NR	2002	Italy	Single centre	Incidence of curative reoperation
	Wille‐Jørgensen et al.^78^	JAMA	COLOFOL	2018	Denmark	Multicenter	OS at 5 years
	Verberne et al.^79^	BJS	CEA watch	2017	The Netherlands	Multicenter	Curable recurrence (proportion)
	Rosati et al.^80^	Ann Oncol	NR	2016	Italy	Multicenter	OS at 8 years

Abbreviations: DFS, disease‐free survival; LRR, locoregional recurrence; NR, not reported; OS, overall survival; TME, total mesorectal excision; PFS, progression‐free survival; hPFS, hepatic progression‐free survival; ypCR, pathologic complete response; pNC/CR, pathologic near complete or complete response; LRR, locoregional recurrence rate.

**FIGURE 3:**
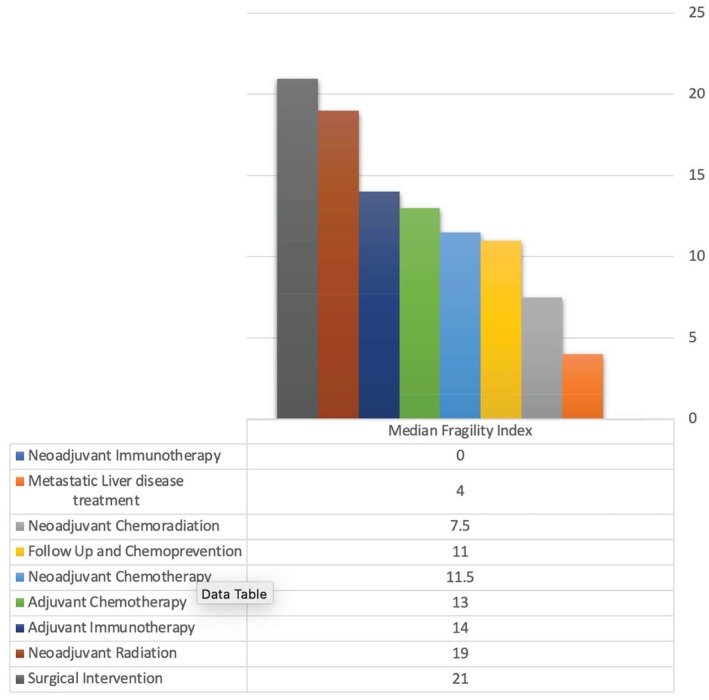
Fragility Index for all included studies categorized by treatment type.

**FIGURE 4:**
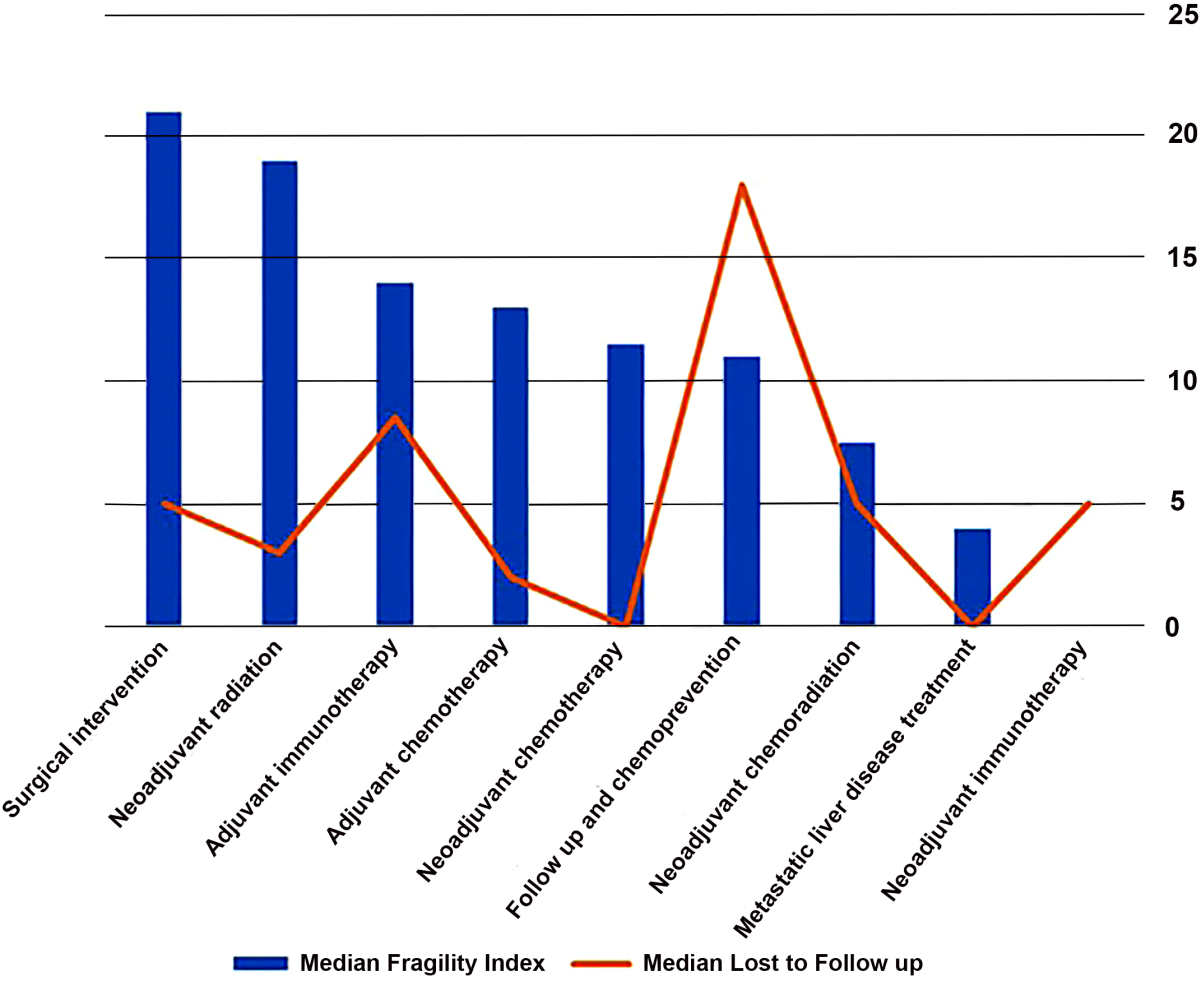
Correlation between the median Fragility Index and the median number of patients lost to follow‐up of included studies based on type of treatment.

Overall, the median FI for all studies was 9, with an interquartile range (IQR) of 2–20 (Figure 3). Surgical Intervention studies had the highest median FI of 21 (IQR 7–27), followed by neoadjuvant radiotherapy studies with a median of 19 (IQR 14–25). Adjuvant chemotherapy studies followed with a median FI of 13 (IQR 6–19.5), while adjuvant immunotherapy studies had a median of 14. Neoadjuvant chemotherapy therapy studies showcased a median of 11.5 (IQR 0–22), while follow‐up and chemoprevention studies had a median of 11 (IQR 2–24). Metastatic liver disease treatment studies had a median of 4 (IQR 0–8). Neoadjuvant immunotherapy studies had the lowest median FI of 0, indicating the least robust evidence among the treatment types (Figure 3). The median number of patients lost to follow‐up was 2 (IQR 0–14). The median FI exceeded the median number of patients lost to follow‐up in the surgical intervention studies (21 vs. 5), adjuvant chemotherapy (13 vs. 2), neoadjuvant radiotherapy (19 vs. 3), neoadjuvant chemotherapy (12 vs. 0), neoadjuvant chemoradiation (7.5 vs. 5), adjuvant immunotherapy (14 vs. 8.5) and metastatic liver disease treatment (4 vs. 0), indicating robust evidence. However, for follow‐up and chemoprevention (11 vs. 18), and neoadjuvant immunotherapy (0 vs. 5), the FI was lower, suggesting potential vulnerabilities in these treatments' study outcomes (Figure 4).

## 4 DISCUSSION

Our study assessed the robustness of RCTs used in the formulation of treatment guidelines for rectal cancer using the FI as a measure of the reliability of results. Our findings indicate that most treatment types included in the NCCN rectal cancer guidelines are based on robust evidence. Not surprisingly, the most recent type of therapy introduced into the guidelines, neoadjuvant immunotherapy, had the weakest FI as compared to other treatments.

RCTs have long been considered as the cornerstone of evidence‐based medicine. However, recent literature has drawn attention to the limitations of using *p*‐values to report statistical significance of outcomes in clinical trials.^81^ The reliance on small *p*‐values, often without considering the practical significance or robustness of the findings, has been a major point of criticism.^6^

Given the limitations associated with *p*‐values, there is a growing need for alternative tools to evaluate the robustness of RCTs. One such tool is the FI, which quantitatively estimates the vulnerability of trial results.^82^ The FI defines the minimum number of patients whose status would need to convert from event to nonevent to render a statistically significant result nonsignificant. FI can enhance clinical trial interpretation by providing a more nuanced assessment of the robustness of study results. While *p*‐values indicate statistical significance, the FI quantifies how fragile the findings are to minor data changes, offering a more comprehensive view of trial reliability. Incorporating FI into clinical practice could guide treatment decisions by highlighting the strength of evidence supporting specific interventions.^83^ Additionally, FI could be used in meta‐analyses to assess the consistency of findings across studies and help refine clinical guidelines. Training researchers and clinicians to consider FI alongside traditional metrics could foster a more critical evaluation of clinical trial data, ensuring that treatment recommendations are based on robust and reproducible evidence.^84^

At present, there is no universally agreed‐upon value for the FI that denotes it as “strong” or “weak.” However, the correlation between the significance of the p value and the FI cannot be disputed. For example, a FI of 8 correlates with a *p*‐value under .004, indicating that a larger number of patients (= 8) needed to move from “event” to “nonevent” for the result to become nonsignificant, while studies with an outcome manifested as *p*‐value <.05 will only require one or two patients for the study to lose statistical significance.^85^ In addition, the results of a trial should be viewed with particular skepticism if the number of patients who are lost to follow‐up is greater than the FI given that the unknown outcomes of these patients could alter the results.^86^ It is crucial to approach trial results with caution, especially if the number of patients who did not complete the trial surpasses the FI.

Several studies used the FI to critique oncologic research. For instance, a cross‐sectional study evaluating Phase 3 RCTs of immune checkpoint inhibitors suggested that many of these trials had a low FI for OS, resulting in uncertainty regarding their potential clinical benefit.^87^ A study assessing the robustness of RCTs regarding the treatment of cholangiocarcinoma (CC) found that the RCTs showed a low degree of robustness with a frequent proportion of associated spin.^88^ Another study revealed that the statistical significance of RCTs in common solid tumors can be reversed often with a small number of additional events.^89^

The FI has also been used to assess treatment guidelines. A study evaluating RCTs supporting the American College of Cardiology (ACG) guidelines for myocardial revascularization found that over 40% of trials revealed an FI that was lower than the number of patients lost to follow‐up.^9^, ^83^ Another study analyzing the strength of data supporting the ACG guidelines for ‘Management of Crohn's disease in adults’ found that the majority of the RCTs relied on a small number of superior events for statistical significance, thus questioning the validity of their conclusions.^90^

While our study provides valuable insights into the robustness of RCTs, it is not without limitations. First, some of the included studies, were referenced in the NCCN rectal cancer guidelines as part of broader treatment strategies, although rectal cancer was not the focus of their investigation.^50^, ^51^ Although these studies were included due to their citation in the guidelines, their findings may not be specific to rectal cancer. Additionally, some of the included studies investigated colorectal cancer, encompassing both colon and rectal cancer. This broader focus may affect the precision of the findings, as outcomes and treatment responses could differ between colon and rectal cancer. These factors should be taken into account when interpreting the results of our study. In addition, the FI, while a useful tool, simplifies the complex relationships between sample size, effect size, and *p*‐value. The applicability of the FI is confined to dichotomous outcomes and cannot be used to examine noninferiority studies.^86^ Furthermore, the absence of a universally recognized threshold for the FI makes it challenging to definitively categorize a study as either “fragile” or “robust.” However, when comparing the median FI of the studies included in the NCCN guidelines for the treatment of rectal cancer to other assessments of randomized controlled trials previously published, the result is much higher in the NCCN guidelines, which is a strong indicator of the robustness of evidence in the trials included within these guidelines.

## 5 CONCLUSION

Our study found that the FI in the NCCN guidelines is high, suggesting that the evidence supporting the treatment recommendations of the NCCN guidelines is robust. However, it is essential to recognize that while RCTs are pivotal in evidence‐based medicine, the robustness of many trials can be questionable. Tools like the FI provide an additional means of assessing and communicating the strength of statistical conclusions, emphasizing the need for a more comprehensive approach to evaluating trial results beyond just *p*‐values.

## AUTHOR CONTRIBUTIONS

**Nir Horesh:** Conceptualization; investigation; methodology; validation; visualization; writing – original draft; formal analysis; data curation. **Justin Dourado:** Conceptualization; investigation; writing – original draft; methodology; validation; visualization; formal analysis; data curation. **Peter Rogers:** Investigation; writing – review and editing; data curation; formal analysis. **Pauline Aeschbacher:** Investigation; data curation; writing – review and editing; formal analysis. **Zoe Garoufalia:** Investigation; data curation; writing – review and editing; formal analysis. **Rachel Gefen:** Investigation; data curation; writing – review and editing; formal analysis. **Ebram Salama:** Investigation; data curation; writing – review and editing; formal analysis. **Sameh Hany Emile:** Investigation; data curation; formal analysis. **Steven D. Wexner:** Conceptualization; project administration; supervision; writing – review and editing.

## CONFLICT OF INTEREST STATEMENT

Dr. Wexner is a consultant for ActivSurgical, Baxter, Becton, Dickinson and Co., Intuitive Surgical, OstomyCure, Takeda, and Virtual Ports, is chair of the Data Safety Monitoring Board of Polypid, has consulting agreements with stock options for consulting with GI View, OstomyCure, and Virtual Ports, and receives royalties from Intuitive Surgical, Karl Storz Endoscopy America Inc., and Unique Surgical Solutions, LLC. Dr. Emile is a consultant for Becton, Dickinson and Co. None of the other authors report any financial disclosures or potential conflicts of interest.

## Data Availability

The data that support the findings of this study are available from the corresponding author upon reasonable request.
